# Effects of Substitution of Wheat Straw by Giant Reed on Growth Performance, Serum Biochemical Parameters, Nutrient Digestibility, and Antioxidant Properties of Sheep

**DOI:** 10.3390/ani14243678

**Published:** 2024-12-20

**Authors:** Kai Zhang, Yibo Yan, Rui Zhao, Xianyi Song, Liying Du, Bochi Zhang, Chunlei Yang, Xiaopeng Tang

**Affiliations:** 1 College of Animal Science, Shanxi Agricultural University, Taiyuan 030032, China206434yyb@sxau.edu.cn (Y.Y.); zr734948896@163.com (R.Z.); sxnysxy@163.com (X.S.); duliying@sxau.edu.cn (L.D.); 15035182928@163.com (B.Z.); wkyhlycl@126.com (C.Y.); 2 State Engineering Technology Institute for Karst Desertfication Control, School of Karst Science, Guizhou Normal University, Guiyang 550025, China

**Keywords:** antioxidant capacity, giant reed, growth performance, nutrient digestibility, unconventional forage resources, sheep

## Abstract

The development and utilization of unconventional forage resources is crucial to alleviating the current situation of shortage of forage resources. Giant reed (*Arundo donax*) is a promising forage resource from the Poaceae family, one of the largest herbaceous plants globally, with fast growth, high biomass yield, and strong ecological adaptability. However, there are still very few reports on the use of giant reed in livestock and poultry production. The purpose of this study was to evaluate the effects of adding giant reed instead of wheat straw in total mixed ration (TMR) diets on growth performance, blood biochemical indexes, nutrient digestibility, and antioxidant properties of sheep, thereby providing a theoretical basis for the development and utilization of giant reed herbage resources. A total of 24 fattening sheep (Han × Duper) with similar body weight (20 kg), age (2 months), and health status were randomly divided into four groups with six replicates per group. Sheep in the control group were fed a basal diet (CON), and those in the experimental groups were fed giant reed Lvzhou No. 1 instead of wheat straw, with replacement proportions of 10% (GR10), 20% (GR20), and 30% (GR30) of the total diet, respectively. The results showed that (1) the body weight (FBW) and average daily gain (ADG) of sheep in the GR20 and GR30 groups were higher than those of sheep in the CON and GR10 groups (*p* < 0.05). Meanwhile, the feed to gain ratio (F/G) of sheep in the GR20 and GR30 groups was lower than those sheep in the CON and GR10 groups (*p* < 0.05), and the F/G of the GR30 group was lower than that of the GR20 group (*p* < 0.05). (2) The apparent digestibility of DM and CP in groups GR10, GR20 and GR30 was significantly higher than that in group CON (*p* < 0.005). The digestibility of NDF and ADF in groups GR20, and GR30 was significantly higher than that in the CON and GR10 groups (*p* < 0.05). (3) dietary substitution of giant reed for wheat straw had no effect on serum biochemical indices, except serum glucose (GLU, *p* = 0.014) of sheep. In addition, the substitution of giant reed for wheat straw had a tendency to decrease serum urea content of sheep (*p* = 0.098). (4) Dietary substitution of giant reed for wheat straw significantly improved serum T-SOD (*p* < 0.001) and T-AOC (*p* < 0.001), and significantly decreased MDA (*p* < 0.001) of sheep. In conclusion, replacing wheat straw with giant reed can significantly enhance growth performance, nutrient digestibility, and antioxidant capacity in sheep without adverse effects on their normal physiological functions.

## Simple Summary

Giant reed (*Arundo donax*) is a promising forage resource that offers high yield, rapid growth, and good nutritional value. However, the utilization of giant reed in animal production remains limited. This study aimed to assess the effects of giant reed (Lvzhou No. 1) as a substitute for wheat straw. Specifically, it examined growth performance, nutrient digestibility, serum biochemical parameters, and antioxidant properties in sheep. The findings indicate that substituting wheat straw with giant reed significantly enhances growth performance, nutrient digestibility, and antioxidant capacity in sheep without compromising their normal physiological functions.

## 1. Introduction

With the development of animal husbandry, the demand for feedstuff is increasing, and the contradiction of “competition for food between humans and animals” is becoming increasingly prominent in China, which seriously restricts the sustainable development of animal husbandry [1,2]. China is rich in plant resources, many of which have the potential to be used as feed materials [3]. The rational development and utilization of unconventional feed resources, such as herbaceous plants, are of great significance to alleviate the current situation of feed resource shortage. Therefore, some plants with large biomass, fast growth rates, and balanced nutrition, such as *Pennisetum* sp. [3] and giant reed (*Arundo donax*) [4], have gradually entered people’s attention as feed resources.

Giant reed (*Arundo donax*), a perennial plant belonging to the Poaceae family, ranks among the largest herbaceous plants globally [5]. Predominantly found in tropical and subtropical regions, giant reed exhibits robust reproductive performance, a fast growth rate, a high biomass yield, and strong ecological adaptability [6]. Consequently, the application range of giant reed is extensive. Traditionally, the giant reed has been utilized for making walking sticks, lattices, baskets, fences, fishing rods, musical instruments, and construction materials [7]. Nowadays, its high biomass productivity and low prices render it a highly attractive lignocellulosic raw material for industrial bioproducts, including papermaking, industrial enzymes, and fuel ethanol production [6,8,9,10]. With strong adaptability and stress resistance, it serves as a pioneer plant for restoring vegetation on wastelands, preventing erosion, and consolidating soil, thereby playing a vital role in environmental management [11,12]. Furthermore, giant reed has demonstrated potential medicinal applications in treating acute upper respiratory tract infection, suppurative tonsillitis, and high fever due to its capacity to inhibit biofilm growth [10,13]. Additionally, the tender, green, and juicy, delicate branches and leaves of giant reed have a high crude protein content, and its yield, dry matter, and nutrient content remain stable across various ecological environments, making it a promising feed resource for livestock [4,14]. However, there are still very few reports on the use of giant reed in livestock and poultry production. It is crucial to investigate its feeding effects if it is to be used as a feed resource.

The advantages of giant reed as a potential feed resource lie in its large biomass, rich nutrition, and rapid growth rate. Studies have shown that the dry weight yield of the second- or third-year giant reed could reach 45–50 tons/ha [15], and the fresh weight yield of giant reed could reach 192.75 tons/ha in soil and water loss area [16]. Some studies have shown that giant reed has a high protein content and moderate fiber content, making it a high-quality feed raw material. In particular, the young branches and leaves of giant reed are favored as green feed by animals [4,14]. However, there were significant differences in the nutritional value of different parts and different growth heights of giant reed. For instance, Li et al. [17] showed that the crude protein (CP) content of the leaves, stems, and whole plant of giant reed with a height of 0.5 m was 20.92%, 8.00%, and 16.62%, respectively; and the CP content of the whole plant of giant reed with a height of 1.0 m, 1.2 m, 1.4 m, 1.6 m, and 1.8 m were 12.27%, 12.85%, 13.06%, 11.33%, and 9.21%, respectively. These indicated that the rational development and utilization of giant reed is expected to alleviate the pressure of high-quality forage shortage in China due to its high yield and feeding value.

However, as a feed material for livestock and poultry, giant reed needs to be tested by feeding experiments. In view of the fact that the application of giant reed in livestock and poultry production is still very rare, the main purpose of this study was to evaluate the effects of giant reed (Lvzhou No. 1) as a substitute for wheat straw in total mixed ration (TMR) diets. Specifically, it examined growth performance, blood biochemical indexes, nutrient digestibility, and antioxidant properties to test its feeding effect on sheep, thereby providing a theoretical basis for the development and utilization of giant reed herbage resources.

## 2. Materials and Methods

### 2.1. Giant Reed

In this study, *Arundo donax* cv. Lvzhou No. 1 (Lvzhou No. 1), a new variety of giant reed cultivated by China National Engineering Research Center of JUNCAO Technology (Fujian, China), was planted in the LVzhou No. 1 planting demonstration base in Yaofeng Town, Yuncheng, China (111°13′59.365″ E, 35°9′19.030″ N). This area has a continental semi-humid monsoon climate with four distinct seasons, a mild climate, an average annual temperature of 12.8 °C, a frost-free period of 205 days, annual rainfall of 506 mm, and an annual sunshine duration of 2293 h. The giant reed used in this experiment was collected when the plant height was 1.2 m to 1.4 m, and its conventional nutrients crude protein (CP), crude fat (EE), and ash were determined following the method of AOAC (1995) [18], while the acid detergent fiber (ADF) and neutral detergent fiber (NDF) were determined using the technique described by Van Soest et al. (1991) [19]. The conventional nutrients of giant reed are shown in Table 1.

### 2.2. Animals and Diets

The experiment was conducted at Xiaxian Kaixin Animal Husbandry Development Co., Ltd. (Yuncheng, China). A total of 24 fattening sheep (Han × Duper) with similar body weight (20 kg), age (2 months), and health status were randomly divided into 4 groups with 6 replicates per group. The experimental groups were fed giant reed Lvzhou No. 1 instead of wheat straw, with replacement proportions of 10% (GR10), 20% (GR20), and 30% (GR30) of the total diet, while the control group was fed a basal diet (CON) (Table 2). The experimental diet followed the meat sheep breeding standard recommended by NRC (2007) [20] with a daily weight gain target of 200 g/d. The composition and nutritional levels of the diet are shown in Table 3. The experimental sheep were individually housed in group feeding facilities that were cleaned, disinfected, and subjected to unified deworming and immunization before feeding. They were fed twice daily (07:30 and 17:00), with access to free drinking water and regular lighting. The experiment lasted for 60 days, with a 15-day pre-trial period and a 45-day trial period.

### 2.3. Growth Performance

Before each feeding, the remaining feed in the tank was cleared, the feeding amount and remaining amount were accurately weighed and recorded, and the daily feed intake of each test sheep was calculated and recorded. At 07:00 on the 1st and 45th day of the trial period, the test sheep were weighed on an empty stomach, and the average daily gain (ADG), average daily feed intake (ADFI), and feed to gain ratio (F/G) were calculated.

### 2.4. Nutrient Apparent Digestibility

Feed samples were collected by day 1 and day 40 of the experiment. On days 40–44 of the experiment, each group began to collect feces incompletely. Then, 10% of the total fecal samples were subsampled, and 10 mL of HCl (10%) was added to prevent ammonia loss. The feed and fecal samples were dried at 65 °C for 48 h, moistened for 24 h, and passed through a 0.5 mm screen for subsequent analysis. Dry matter (DM) and CP in feed and fecal samples were determined following the method of AOAC (1995) [18], while the ADF and NDF were determined using the technique described by Van Soest et al. (1991) [19]. The apparent digestibility was calculated using the following equation:

Apparent digestibility (%) = [(Nutrient in feed − Nutrient in feces)/Nutrient in feed] × 100

### 2.5. Serum Biochemical Parameters

At the end of the experiment, blood was collected from the jugular vein of 6 sheep in each group after 12 h of fasting. After standing for 30 min, the supernatant was centrifuged at 2500× *g* for 15 min. The supernatant was divided into Eppendorf tubes and stored at −20 °C. Serum biochemical indexes of aspartate aminotransferase (AST), alanine aminotransferase (ALT), alkaline phosphatase (ALP), total protein (TP), albumin (ALB), urea, cholesterol (TC), glucose (GLU), and triglyceride (TG), high-density lipoprotein (HDL), and low-density lipoprotein (LDL) were detected by Mindray automatic biochemical analyzer BS-200 (Shenzhen Mindray Bio-Medical Electronics Co., Ltd., Shenzhen, China) in accordance with the manufacturer’s instructions.

### 2.6. Serum Antioxidant Properties

Serum samples were obtained as above. Total superoxide dismutase (T-SOD), malondialdehyde (MDA), and total antioxidant capacity (T-AOC) were measured using SOD assay kit (A001-3), MDA assay kit (A003-2), and T-AOC assay kit (A015-1), respectively, respectively, according to the instructions of the manufacturer (Nanjing Jiancheng Bioengineering Institute, Nanjing, China).

### 2.7. Statistical Analysis

The experimental data were performed using Shapiro–Wilk of IBM SPSS statistics 21.0 (SPSS, Inc., Chicago, IL, USA) to test data of normal distribution, and *p* > 0.05 was considered the data are normally distributed. Then, data were statistically analyzed using one-way ANOVA procedure by using the IBM SPSS statistics 21.0 software (SPSS, Inc. Chicago, IL, USA). Significant differences among the means were determined by Duncan’s multiple comparison, and linear effect analysis was performed on the replacement level of giant reed. The data are presented as the means ± SEM. A *p*-value of < 0.05 was considered statistically significant.

## 3. Results

### 3.1. Effects of Different Proportions of Giant Reed on Growth Performance of Sheep

As shown in Table 4, dietary substitution of giant reed for wheat straw had significant effects on final body weight (FBW), ADG, and F/G of sheep in the whole experimental period (*p* < 0.001). The FBW and ADG of sheep in the GR20 and GR30 groups were higher than that of sheep in the CON and GR10 groups (*p* < 0.05). Meanwhile, the F/G of sheep in the GR20 and GR30 groups was lower than those of sheep in the CON and GR10 groups (*p* < 0.05), and the F/G of the GR30 group was lower than that of the GR20 group (*p* < 0.05). Dietary substitution of giant reed for wheat straw had no effect on ADFI of sheep. Linear analysis showed that the FBW (*p* < 0.001) and ADG (*p* < 0.001) of sheep were linearly increased, and the F/G (*p* < 0.001) of sheep was linearly decreased with the increase in the substitution amount of giant reed. The increase in ADG and decrease in F/G in GR30 indicated a potential economic benefit of replacing wheat straw with giant reed.

### 3.2. Effects of Different Proportions of Giant Reed on Nutrient Apparent Digestibility of Sheep

As can be seen from Table 5, dietary substitution of giant reed for wheat straw had significant effects on the apparent digestibility of DM (*p* < 0.001), CP (*p* = 0.011), NDF (*p* < 0.001) and ADF (*p* = 0.032). The apparent digestibility of DM and CP in the GR10, GR20, and GR30 groups was significantly higher than that in the CON group (*p* < 0.005), while there was no significant difference between the GR20 and GR30 groups and significantly higher than that in group GR10 (*p* < 0.05). The digestibility of NDF in the GR20 and GR30 groups was significantly higher than that in the CON group (*p* < 0.05), but there was no significant difference between the CON and GR10 groups. The apparent digestibility of ADF in the GR30 group was significantly higher than that in other groups (*p* < 0.05). It showed that the apparent digestibility of DM (*p* < 0.001), CP (*p* < 0.001), and NDF (*p* < 0.001) of sheep was linearly increased with the increase in the substitution amount of giant reed and showed an increasing trend with the increase in the proportion of giant reed in feed (*p* = 0.054). The increase in apparent digestibility of nutrients suggested that giant reed could improve the digestibility and utilization of nutrients in sheep, which was very beneficial to improve the economic benefit of breeding in intensive farming conditions.

### 3.3. Effects of Different Proportions of Giant Reed on Serum Biochemical Parameters of Sheep

Dietary substitution of giant reed for wheat straw had no effect on serum biochemical indices such as TP (*p* = 0.827), ALB (*p* = 0.493), TC (*p* = 0.105), HDL (*p* = 0.0.378), LDL (*p* = 0.193), TG (*p* = 0.602), AST (*p* = 0.464), ALT (*p* = 0.355), and ALP (*p* = 0.488), except serum glucose (GLU, *p* = 0.014) (Table 6). In addition, the substitution of giant reed for wheat straw had a tendency to decrease serum urea (*p* = 0.098). Serum GLU content of sheep in GR30 group is significantly higher than that of CON and GR10 groups (*p* < 0.05), but no significant difference was found between the GR30 group and the GR20 group. There was no significant difference in serum GLU contents among the CON, GR10, and GR20 groups. Linear analysis showed dietary replacement of wheat straw with giant reed can linearly increase serum GLU content (*p* = 0.026) and linearly decrease serum urea content (*p* = 0.024) in sheep.

### 3.4. Effects of Different Proportions of Giant Reed on Serum Antioxidant Properties of Sheep

As shown in Table 7, dietary substitution of giant reed for wheat straw significantly improved serum T-SOD (*p* < 0.001) and T-AOC (*p* < 0.001), and significantly decreased MDA (*p* < 0.001) of sheep. The serum T-SOD and T-AOC of sheep in the GR10, GR20, and GR30 groups were significantly higher than those in the CON group (*p* < 0.05), while the MDA content in the GR20 and GR30 groups was significantly lower than that in the CON and GR10 groups (*p* < 0.05). Notably, the GR30 group showed the best serum antioxidant properties of sheep because it had the highest T-SOD (*p* < 0.05) and T-AOC (*p* < 0.05) activity and the lowest MDA content (*p* < 0.05) compared to the other groups. Linear analysis showed that the serum T-SOD (*p* < 0.001) and T-AOC (*p* < 0.001) of sheep were linearly increased, and the serum MDA (*p* < 0.001) was linearly decreased with the increase in the substitution amount of giant reed. Improvements in antioxidant properties suggested that giant reed may enhance the oxidative stress response in sheep, which could be beneficial in intensive farming conditions.

## 4. Discussion

### 4.1. Effects of Different Proportions of Giant Reed on Growth Performance of Sheep

The feeding experiment of fattening sheep proved that adding giant reed (Luzhou No. 1) raw material to TMR diet could significantly increase the FBW and ADG and significantly reduce F/G but had no effect on ADFI of fattening sheep. The reason may be that giant reed (Luzhou No. 1) exhibits characteristics such as tenderness, juiciness, high protein content, low levels of ADF and NDF, good palatability, and high acceptance by sheep, making it a high-quality roughage material. Compared to wheat straw [21], giant reed had higher CP content (17.45% vs. 4.37%), lower ADF (39.51% vs. 40.96%), and lower NDF (64.91% vs. 66.73%) content. This composition of giant reed may contribute to improved growth performance and overall body health.

The improvement of animal production performance is an important prerequisite for raising economic benefits in livestock and poultry breeding. The results of the present study indicated that replacing 2/3 (GR20) or all (GR30) wheat straw with giant reed in the TMR diet can promote the growth performance of sheep, which is similar to the results of Al Suwaiegh et al. [4], who reported that replacing alfalfa hay with 30.0% giant reed can improve the growth performance and feed efficiency of Aldi goats. In addition, due to its high biomass yield and crude protein content, reasonable price, and availability, giant reed has potential economic benefits for sheep production.

### 4.2. Effects of Different Proportions of Giant Reed on Nutrient Apparent Digestibility of Sheep

Nutrient apparent digestibility refers to the proportion of the diet digested and absorbed in the animal body and is one of the important indexes to measure the nutritional value of the diet [22]. The efficiency of digestion and absorption of dry matter and nutrients also determines the growth performance of animals [22,23]. In this study, the apparent digestibility of nutrients in the TMR diet was enhanced by replacing wheat straw with giant reed, which contributed to the improved growth performance of sheep. The results of the study showed that the apparent digestibility of DM and CP in the GR20 and GR30 groups was significantly higher than that in the CON and GR10 groups. Additionally, the digestibility of NDF and ADF in the GR30 group was significantly higher than that in the other three groups. These findings suggest that replacing wheat straw with giant reed 100% can greatly increase the nutrient digestibility in sheep. This may be due to the fact that giant reed is a tender and succulent green feed; replacing wheat straw with giant reed improves the palatability of TMR diet, and the ratio of digestible cellulose to total fiber in the diet is higher than that of wheat straw, resulting in better digestive characteristics [4,14,21]. Moreover, the nutrients in giant reed are more easily digested and utilized by microorganisms, which promotes the growth and metabolism of rumen microorganisms, and improves the rumen environment, thereby improving the digestibility of the rumen of sheep [14]. For these reasons, the substitution of giant reed for wheat straw showed higher apparent digestibility of nutrients, which is beneficial to the high-efficiency transformation and rapid deposition of nutrients in sheep. Therefore, it can be stated that enhancing nutrient digestibility is a key factor in substituting giant reed for wheat straw to enhance sheep growth performance.

### 4.3. Effects of Different Proportions of Giant Reed on Serum Biochemical Parameters of Sheep

Serum biochemical indexes are often used as important indexes to evaluate the nutrition level, health level, and production performance of animals [24]. For instance, serum urea is one of the important products of protein catabolism, which reflects the metabolism of protein [25]. The decrease in serum urea content indicates that the utilization rate of nitrogen is improved. In the present study, dietary substitution of giant reed for wheat straw linearly decreased serum urea content, in which the GR30 group had the lowest serum urea content, which was consistent with the increase in CP apparent digestibility, suggesting that replacing wheat straw with giant reed improved dietary protein and amino acid availability in sheep. This could be attributed to the promotion of microbial protein synthesis in the sheep rumen by substituting giant reed for wheat straw, resulting in low degradable protein content in the rumen, a good balance of energy and nitrogen, and a well-maintained balance of amino acids in sheep metabolism. GLU is a vital nutrient for maintaining animal life and plays a crucial role in animal body composition and energy metabolism [26]. The serum GLU is mainly derived from the decomposition of liver glycogen and propionic acid produced by the decomposition of dietary carbohydrates by rumen microorganisms, serving as the main energy source for the body, and its level reflects the carbohydrate metabolism and homeostasis of the animal body [27]. In the present study, 100% replacement of wheat straw with giant reed (the GR30 group) significantly increased the serum GLU content of sheep. The increase in serum glucose concentration by dietary addition of giant reed instead of wheat straw may be due to the fact that giant reed is a fresh and juicy green feed, and the proportion of soluble carbohydrates and digestible cellulose in the diet is higher than that of wheat straw, which improves the digestibility of carbohydrates of sheep, thus increasing the glucose absorbed by the intestine and ultimately leading to a higher serum glucose content. The higher apparent digestibility of ADF and NDF in this study also proved that the substitution of giant reed for wheat straw improved the carbohydrate digestion of sheep. However, the present study showed that the substitution of asparagine for wheat straw had no effect on other serum biochemical indexes such as TP, ALB, urea, TC, HDL, LDL, TG, AST, ALT, and ALP. Therefore, the results of the present study suggested that the substitution of giant reed for wheat straw had no negative effect on the normal physiological indexes but can improve energy and protein metabolism of sheep.

### 4.4. Effects of Different Proportions of Giant Reed on Serum Antioxidant Properties of Sheep

The antioxidant system helps prevent damage from free radicals produced during metabolic processes and environmental stimuli, enhancing immune response and improving disease resistance, thereby improving the growth performance of animals [28,29]. SOD serves as the primary defense in the endogenous antioxidant system, eliminating free radicals, converting O^2−^ to H_2_O_2_, breaking down superoxide, and shielding cells and tissues from oxidative harm [30]. T-AOC represents the overall level of antioxidant capacity of the body [31]. MDA is the main product of lipid peroxidation, which indirectly reflects the degree of oxidative damage of animal cells [32]. In the process of livestock and poultry breeding, many factors can directly or indirectly cause oxidative damage to the animal body itself, resulting in lower feed conversion rates and lower growth performance, resulting in increased breeding costs [33]. Therefore, the improvement of animal antioxidant performance is directly related to the improvement of animal production performance. The present study showed that the substitution of giant reed for wheat straw significantly improved the antioxidant capacity of sheep, as evidenced by increased serum T-SOD and T-AOC and decreased serum MDA level. This may be related to the abundance of indoles alkaloids in giant reed, which has a variety of biological functions, such as anti-inflammation and anti-tumor properties [34,35]. Among these, gramine (N, N-dimethyl-3-amino-methylindole) is the primary component of the alkaloid, a well-known natural product widely utilized in medicine [36]. Studies have shown that giant reed and its derivatives have obvious biological functions such as antiviral [37], antibacterial [38], antifungal [39], antioxidation [40], anti-inflammatory [41], antitumor [42], and activation of 5-HT receptors [43]. It is precisely because of the existence of active substances such as asparagine that the antioxidant level of sheep is improved, thus improving the growth performance of sheep.

In conclusion, the improvement of growth performance of sheep by substituting giant reed for wheat straw may be related to the fact that giant reed can improve nutrient apparent digestibility, improve serum metabolic indexes, and enhance the antioxidant capacity of sheep. Therefore, this study confirmed that giant reed can be used as roughage for ruminants such as sheep and bring economic benefits to livestock production. First of all, giant reed has a large biomass, moderate nutrition, a suitable price, and, more importantly, is easy to obtain, which can not only solve the problem of shortage of feed resources in China but also save the transportation cost of areas lacking straw resources such as wheat and corn. Secondly, giant reed has developed roots and strong stress resistance, which can be used as a pioneer plant in the control of rocky desertification in karst areas and has important ecological benefits. At the same time, the cultivated giant reed can be used for cattle and sheep feeding, which can increase farmers’ income and have good social benefits. Therefore, giant reed is a kind of high-quality grass seed with economic, social, and ecological benefits. However, the application of giant reed in livestock and poultry production is still less, and this study only discusses its application effect in sheep production, which is far from enough for the use of giant reed as a conventional feed resource. In order to promote the resource utilization of giant reed feed, future research can focus on improving the feeding value of giant reed, such as silage, fermentation, etc., and also discuss the effect of mixed feeding with other feed materials.

## 5. Conclusions

Replacing wheat straw with giant reed can significantly enhance growth performance, nutrient digestibility, and antioxidant capacity in sheep without adverse effects on their normal physiological functions. Giant reed (Lvzhou No. 1) shows high feeding value and has potential for conventional feed resources. However, the present study has some limitations; for example, the animal experiment time is too short, and the feeding effect of different stages of sheep growth is not involved. The mechanism of the improvement of animal performance by giant reed has not been discussed in detail. Further studies on the mechanism of improving the performance of sheep at different stages may be considered in the future. In short, the sustainable development of giant reed as a forage resource can help alleviate the shortage of forage resources in China, bringing about positive economic, social, and ecological benefits.

## Figures and Tables

**Table 1:** Conventional nutrients of giant reed (DM basis, %).

Items	Content
Crude protein	17.45
Crude fat	1.78
Ash	10.71
Acid detergent fiber	39.51
Neutral detergent fiber	64.91

**Table 2:** Experimental groups design.

Experimental Groups	Diet Composition
CON	Concentrate: Wheat straw = 7:3
GR10	Concentrate: Wheat straw: Giant reed = 7:2:1
GR20	Concentrate: Wheat straw: Giant reed = 7:1:2
GR30	Concentrate: Giant reed = 7:3

**Table 3:** Experimental diet composition and nutrient levels (air-dried basis, %).

Item	CON	GR10	GR20	GR30 ^3^
Ingredients				
Corn	23	23	23	23
Soybean meal	3.96	3.96	3.96	3.96
Wheat bran	38.94	38.94	38.94	38.94
Wheat straw	30.00	20.00	10.00	0.00
Giant reed	0	10.00	20.00	30.00
Premix ^1^	4.00	4.00	4.00	4.00
Total	100.00	100.00	100.00	100.00
Nutrient level ^2^				
ME, Mcal/kg	2.26	2.37	2.48	2.59
CP	11.23	12.53	13.83	15.13
NDF	44.89	43.89	42.89	41.89
ADF	23.55	22.55	21.55	20.55

^1^ Premix provided the following for per kg of diets: Cu (as copper sulfate) 15 mg, Fe (as ferrous sulfate) 55 mg, Mn (as manganese sulfate) 40 mg, Se (as sodium selenite) 0.3 mg, I (as potassium iodide) 0.5 mg, Co 0.2 mg, VA 20,000 IU, VD 4000 IU, VE 400 IU. ^2^ ME was a calculated value, while the others were measured values. ME, metabolizable energy; CP, crude protein; ADF, acid detergent fiber; NDF, neutral detergent fiber. ^3^ The experimental groups were fed giant reed Lvzhou No. 1 instead of wheat straw, with re-placement proportions of 10% (GR10), 20% (GR20), and 30% (GR30) of the total diet, while the control group was fed a basal diet (CON).

**Table 4:** Effects of different proportions of giant reed on growth performance of sheep.

Item	CON	GR10	GR20	GR30	SEM	*p*-Value	
						*p_T_*	*p_L_*
IBW, kg	20.28	20.32	20.20	20.23	0.02	0.182	0.122
FBW, kg	28.30 ^b^	28.75 ^b^	29.33 ^a^	29.74 ^a^	0.14	<0.001	<0.001
ADG, g/d	178.14 ^b^	187.26 ^b^	202.70 ^a^	211.51 ^a^	3.42	<0.001	<0.001
ADFI, g/d	1059.21	1070.52	1100.55	1087.48	10.94	0.583	0.249
F/G	5.97 ^a^	5.72 ^a^	5.43 ^b^	5.14 ^c^	0.08	<0.001	<0.001

Values are expressed as means ± SEM (*n* = 6). The experimental groups were fed giant reed Lvzhou No. 1 instead of wheat straw, with replacement proportions of 10% (GR10), 20% (GR20), and 30% (GR30) of the total diet, while the control group was fed a basal diet (CON). ^a,b,c^ means that values within a row with different superscript letters were significantly different. *p* < 0.05 was considered statistically significant. *p_T_*: the significance of the difference among treatments; *p_L_*: linear effect significance. IBW: initial body weight, FBW: final body weight, ADG: average daily gain, ADFI: average daily feed intake, F/G: feed to gain ratio.

**Table 5:** Effects of different proportions of giant reed on nutrient apparent digestibility of sheep (%).

Item	CON	GR10	GR20	GR30	SEM	*p*-Value	
						*p_T_*	*p_L_*
DM	52.34 ^c^	56.01 ^b^	59.60 ^a^	60.65 ^a^	0.62	<0.001	<0.001
CP	52.91 ^c^	53.42 ^b^	56.84 ^a^	57.74 ^a^	0.60	0.011	<0.001
NDF	49.72 ^c^	51.62 ^c^	61.46 ^b^	63.47 ^a^	0.74	<0.001	<0.001
ADF	45.95 ^d^	47.09 ^c^	48.13 ^b^	51.65 ^a^	0.88	0.032	0.054

Values are expressed as means ± SEM (*n* = 6). The experimental groups were fed giant reed Lvzhou No. 1 instead of wheat straw, with replacement proportions of 10% (GR10), 20% (GR20), and 30% (GR30) of the total diet, while the control group was fed a basal diet (CON). ^a,b,c,d^ means that values within a row with different superscript letters were significantly different. *p* < 0.05 was considered statistically significant. *p_T_*: the significance of the difference among treatments; *p_L_*: linear effect significance. DM: dry matter, CP: crude protein, NDF: neutral detergent fiber, ADF: acid detergent fiber.

**Table 6:** Effects of different proportions of giant reed on serum biochemical parameters of sheep.

Item	CON	GR10	GR20	GR30	SEM	*p*-Value	
						*p_T_*	*p_L_*
GLU (mmol/L)	4.80 ^b^	4.43 ^b^	4.84 ^ab^	5.38 ^a^	0.11	0.014	0.026
TP (g/L)	55.55	54.33	54.06	56.20	0.88	0.827	0.838
ALB (g/L)	25.62	25.20	25.44	26.93	0.42	0.493	0.277
Urea (mmol/L)	7.64 ^a^	6.17 ^ab^	6.52 ^ab^	5.28 ^b^	0.34	0.098	0.024
TC (mmol/L)	1.49	1.41	1.31	1.40	0.03	0.105	0.124
HDL (mmol/L)	0.73	0.79	0.72	0.77	0.01	0.378	0.775
LDL (mmol/L)	0.61	0.49	0.50	0.55	0.02	0.193	0.470
TG (mmol/L)	0.27	0.31	0.29	0.25	0.01	0.602	0.523
AST (U/L)	82.88	81.6	76.80	76.75	1.68	0.464	0.126
ALT (U/L)	11.97	11.70	10.95	10.92	0.50	0.355	0.098
ALP (U/L)	444.09	437.63	392.79	428.40	12.36	0.488	0.418

Values are expressed as means ± SEM (*n* = 6). The experimental groups were fed giant reed Lvzhou No. 1 instead of wheat straw, with replacement proportions of 10% (GR10), 20% (GR20), and 30% (GR30) of the total diet, while the control group was fed a basal diet (CON). ^a,b^ means that values within a row with different superscript letters were significantly different. *p* < 0.05 was considered statistically significant. *p_T_*: the significance of the difference among treatments; *p_L_*: linear effect significance. GLU: glucose, TP: total protein, ALB: albumin, TC: cholesterol, HDL: high-density lipoprotein, LDL: low-density lipoprotein, TG: triglyceride, AST: aspartate aminotransferase, ALT: alanine aminotransferase, ALP: alkaline phosphatase.

**Table 7:** Effects of different proportions of giant reed on serum antioxidant parameters of sheep.

Item	CON	GR10	GR20	GR30	SEM	*p*-Value	
						*p_T_*	*p_L_*
T-SOD (U/mL)	84.09 ^d^	104.42 ^c^	126.92 ^b^	144.89 ^a^	5.00	<0.001	<0.001
T-AOC (U/mL)	9.10 ^d^	11.43 ^c^	12.99 ^b^	14.73 ^a^	0.45	<0.001	<0.001
MDA (nmol/mL)	4.73 ^a^	4.30 ^a^	3.12 ^b^	2.45 ^c^	0.21	<0.001	<0.001

Values are expressed as means ± SEM (*n* = 6). The experimental groups were fed giant reed Lvzhou No. 1 instead of wheat straw, with replacement proportions of 10% (GR10), 20% (GR20), and 30% (GR30) of the total diet, while the control group was fed a basal diet (CON). ^a,b,c,d^ means that values within a row with different superscript letters were significantly different. *p* < 0.05 was considered statistically significant. *p****_T_***: the significance of the difference among treatments; *p_L_*: linear effect significance. T-SOD: total superoxide dismutase, T-AOC: total antioxidant capacity, MDA: malondialdehyde.

## Data Availability

The data presented in this study are available on request from the corresponding author.
